# Fluid Mechanics of Fetal Left Ventricle During Aortic Stenosis with Evolving Hypoplastic Left Heart Syndrome

**DOI:** 10.1007/s10439-022-02990-5

**Published:** 2022-06-22

**Authors:** Hong Shen Wong, Hadi Wiputra, Andreas Tulzer, Gerald Tulzer, Choon Hwai Yap

**Affiliations:** 1 Department of Bioengineering, Imperial College London, London, UK; 2 Department of Biomedical Engineering, University of Minnesota, Minneapolis, USA; 3 Department of Pediatric Cardiology, Children’s Heart Center Linz, Kepler University Hospital, Linz, Austria

**Keywords:** Fluid mechanics, Fetal heart, Aortic stenosis, Hypoplastic left heart syndrome, Computational fluid dynamics

## Abstract

In cases of fetal aortic stenosis and evolving Hypoplastic Left Heart Syndrome (feHLHS), aortic stenosis is associated with specific abnormalities such as retrograde or bidirectional systolic transverse arch flow. Many cases progressed to hypoplastic left heart syndrome (HLHS) malformation at birth, but fetal aortic valvuloplasty can prevent the progression in many cases. Since both disease and intervention involve drastic changes to the biomechanical environment, *in-vivo* biomechanics likely play a role in inducing and preventing disease progression. However, the fluid mechanics of feHLHS is not well-characterized. Here, we conduct patient-specific echocardiography-based flow simulations of normal and feHLHS left ventricles (LV), to understand the essential fluid dynamics distinction between the two cohorts. We found high variability across feHLHS cases, but also the following unifying features. Firstly, feHLHS diastole mitral inflow was in the form of a narrowed and fast jet that impinged onto the apical region, rather than a wide and gentle inflow in normal LVs. This was likely due to a malformed mitral valve with impaired opening dynamics. This altered inflow caused elevated vorticity dynamics and wall shear stresses (WSS) and reduced oscillatory shear index at the apical zone rather than mid-ventricle. Secondly, feHLHS LV also featured elevated systolic and diastolic energy losses, intraventricular pressure gradients, and vortex formation numbers, suggesting energy inefficiency of flow and additional burden on the LV. Thirdly, feHLHS LV had poor blood turnover, suggesting a hypoxic environment, which could be associated with endocardial fibroelastosis that is often observed in these patients.

**Supplementary Information:**

The online version contains supplementary material available at 10.1007/s10439-022-02990-5.

## Introduction

Hypoplastic left heart syndrome (HLHS) is a complex critical congenital heart disease that can have severe outcomes.^27^ In a subset of HLHS patients, pathogenesis is associated with aortic stenosis during mid-gestation with a normal-sized or dilated heart, which progresses to HLHS by birth.^4,15^ The aortic stenosis causes depressed left ventricular (LV) function and is accompanied by abnormalities such as bidirectional or retrograde transverse aortic arch flow, monophasic mitral valve inflow, left-to-right foramen flow, and bidirectional flow in the pulmonary veins.^15,17^ Natural history studies showed that only 19.4–36% of such patients will be biventricular at birth, and the remaining will have HLHS single ventricular malformation.^11,15,20^ For this reason, these patients are known to have fetal aortic stenosis with evolving HLHS (feHLHS).

In such patients, fetal aortic valvuloplasty (FAV) has shown much promise in reversing the original prognosis and increasing biventricular birth outcomes. FAV involves introducing a needle through the mother's abdomen into the fetal LV and inflating a balloon catheter at the stenotic aortic valve to relieve the stenosis.^16,32^ Two single-centre studies have reported 66–68% biventricular postnatal outcomes in cases with technically successful FAV.^23,30^ Procedural success and biventricular outcome rates have also shown signs of improvement in the later years, suggesting that experience may improve outcomes.^23^

In both critical aortic stenosis and fetal aortic valvuloplasty, there were drastic changes to the biomechanical environment of the heart. During feHLHS, outflow obstruction caused high pressures in the LV, which can be inferred by the maximum instantaneous peak gradient from the aortic valve (AV) or mitral regurgitation (MR) velocities, estimated by the simplified Bernoulli equation.^17,18^ Elevated LV pressure often leads to severe MR, which can cause left atrial pressurization and dilation, and consequently, premature narrowing or closure of the foramen ovale.^31^ Further, LV ejection fraction and myocardial strains are severely depressed.^9^ After the intervention, de-pressurization of the LV can be achieved, decreased LV end-diastolic volume and acute improvements in diastolic function and ventricular strains.^5,9,24,38^ Furthermore, there is evidence that such changes in hemodynamics after FAV, such as that seen aortic arch and foramen ovale, allowed for increased growth of the AV and mitral valves (MV).^11,17,25^ Unfortunately, there is currently little data on the biomechanical environment of feHLHS hearts before or after the intervention.

The above clinical evidence for feHLHS progression to HLHS, and for interventions to reduce risks of this progression, has led to the prevailing thought that the biomechanical stimuli of the heart exert influence on the growth and development of the heart, and can determine morphological outcomes. Large and small animal experiments have corroborated this notion, the occlusion of lamb fetal foramen ovale was shown to lead to the underdevelopment of LV chamber and aortic and mitral valves,^39^ while left atrial ligation in chick embryo has led to hypoplastic LV^29^ and evidence of fibroelastosis.^22^ For this reason, an improved understanding of LV biomechanics during feHLHS may help develop future strategies for better prognosis or to improve intervention outcomes.

Thus, in this study, we conducted a study of the blood flow mechanics in human fetal LVs with feHLHS, *via* subject-specific computational fluid dynamics (CFD) simulations based on 4D echocardiography images and characterized essential differences against those in normal human fetal LVs.

## Methods

### Patient Data Collection

Data for 5 healthy fetuses and 5 feHLHS were prospectively gathered, from the National University Hospital, Singapore, and the Kepler University Hospital, Austria, with ethics approval from both (DSRB protocol number 2014/00056 at the former, and IRB protocol number 1009/2017 at the latter), and written consent from all participants. All feHLHS cases were within the selection criteria for aortic valvuloplasty intervention^1^ and underwent the intervention later, and their characteristics are listed in Table 1. Data for an additional 7 healthy fetal subjects from our previous publication were also analysed.^36^

**Table 1:** Characteristics of aortic stenosis with evolving hypoplastic left heart syndrome (feHLHS) fetal subjects before aortic valvuloplasty, postnatal outcomes of the same subjects and postnatal procedures undertaken.

Case	GA at scan, (week + day)	Brady-cardia	LV throm-bus	Pericardial effusion	Hydrops	Postnatal Circulation	Outcome	Postnatal procedures
feHLHS - 1	22 + 1	Y	N	N	N	BV	Alive	RK, BV-UV conv., NW
feHLHS – 2	22 + 4	N	Y	N	N	UV	Infant death	NW
feHLHS – 3	29 + 1	N	N	N	Y	BV	Alive	RK
feHLHS – 4	29	N	N	N	Y	BV	Alive	RK
feHLHS – 5	31 + 6	N	N	Y	N	BV	Alive	Surg. Valv., Ross

*GA* gestational age, *LV* left ventricle, *BV* biventricular, *UV* univentricular, *RK* Ross-Konno procedure, *BV-UV conv.* biventricular to univentricular conversion, *NW* Norwood procedure, *Surg. Valv.* surgical valvotomy, *Y* yes, *N* no

### Image acquisition and LV reconstruction

Imaging and image processing methods were as established in our previous studies.^13,37^ Briefly, 4D B-mode ultrasound images were acquired using the Vivid-7®, Vivid E9®, or Vivid E95® (GE Healthcare, Chicago, IL, USA) ultrasound system via the Spatio-Temporal Image Correlation (STIC) mode. The STIC sweep took 10–15 s, with an image capture rate of 70–90 frames per second, thus giving 40 volumes for 1 cardiac cycle. Subsequently, images were exported as a stack of 2D cine images with 0.5mm spacing between consecutive planes. The lumen cavity of the left and right ventricle (LV, RV), and left and right atrium (LA, RA) were segmented with a custom-written semi-automatic lazy-snapping algorithm,^14^ reconstructed via Vascular Modeling Toolkit (VMTK, www.vmtk.org), and smoothed with Geomagic Studio (Geomagic Inc., Morrisville, NC, USA).

A validated cardiac motion estimation algorithm was used to track the motion of the patient-specific reconstructed LV, RV and LA endocardial wall over the cardiac cycle.^35^ This algorithm fits a global motion model of spatial b-splines of temporal Fourier onto results of a set of pair-wise image registrations between different time frames, thus enforcing the cyclic nature of motions. Segmentation was performed at a particular time point, as it could be animated with the motion model to all other time points. The stroke volume of cardiac chambers could then be calculated based on their re-animated motions.

### CFD Simulations

Methods for image-based CFD simulation were adapted from our previous studies,^13,37^ and utilized ANSYS FLUENT 2019 R2 (ANSYS Inc., Canonsburg, PA, USA). LV luminal blood space was meshed into at least 1–1.5 million tetrahedral elements, which was shown to exceed mesh convergence requirements.^36^ A user-defined function was used to control the wall motions according to the motion model from motion tracking. Blood viscosity was modelled with the Carreau-Yasuda model as we previously reported,^13^ while density, *ρ*, was prescribed as 1060 kg/m^3^. Dynamic mesh CFD simulations were conducted to solve the 3D Navier Stokes equation with 400 time steps per cardiac cycle for at least three cycles, and only the last cycle was used for analysis to minimize artefacts of the stagnant initial condition. Convergence criteria were set to be less than 10^−4^ for all scaled residuals.

Inlet and outlet boundary conditions were idealized as instantaneous opening or closing of valves, by converting surfaces between openings and walls. When opened, inlets and outlets were prescribed with pressures that were calculated based on a Windkessel lumped parameter model, described below. In the feHLHS cohort, mitral valve insufficiency was modelled by having a regurgitation orifice that was modelled as a permanently open orifice, located within the mitral orifice zone on the model’s surface. The sizes of all the aortic outlet, mitral inlet, and mitral regurgitation orifice were iteratively adjusted until CFD simulations produced valve velocities that matched clinical patient-specific Doppler scans satisfactorily, including aortic outflow velocity, peak E- and A-wave inflow velocities and peak mitral regurgitation velocity. Due to the low resolution of echo images, the valve morphology and orifice shapes and sizes could not be discerned. Orifices were thus approximated to be at the centre of their likely location, for example, the aortic orifice was assumed to be at the centre of the aortic valve, while the mitral regurgitation orifice was assumed to be at the centre of the mitral valve.

### Lumped Parameter Model of Human Fetal Circulation

A Windkessel lumped parameter model was used for ventricular-vascular coupling in our flow simulations. During the coupling of the model to the CFD boundary conditions, the model was run for 10 cardiac cycles to ensure a steady and periodic state was achieved. The lumped parameter model was adapted from Pennati *et al.*^21^ who proposed a methodology for allometric scaling of the lumped parameter from 38 weeks gestational age (GA) to other GA, as briefly explained in supplementary section S1 (which includes Table S1). However, we recalibrated it to fit more recent human fetal clinical measurements,^10,34^ in contrast to some of its original fit to fetal lamb measurements. To do this, all resistances were scaled by an age-dependent factor, while all compliances were scaled by another. The original value of the inertances and valvular dissipative coefficients were retained from Pennati *et al.*^21^

**Figure 1:**
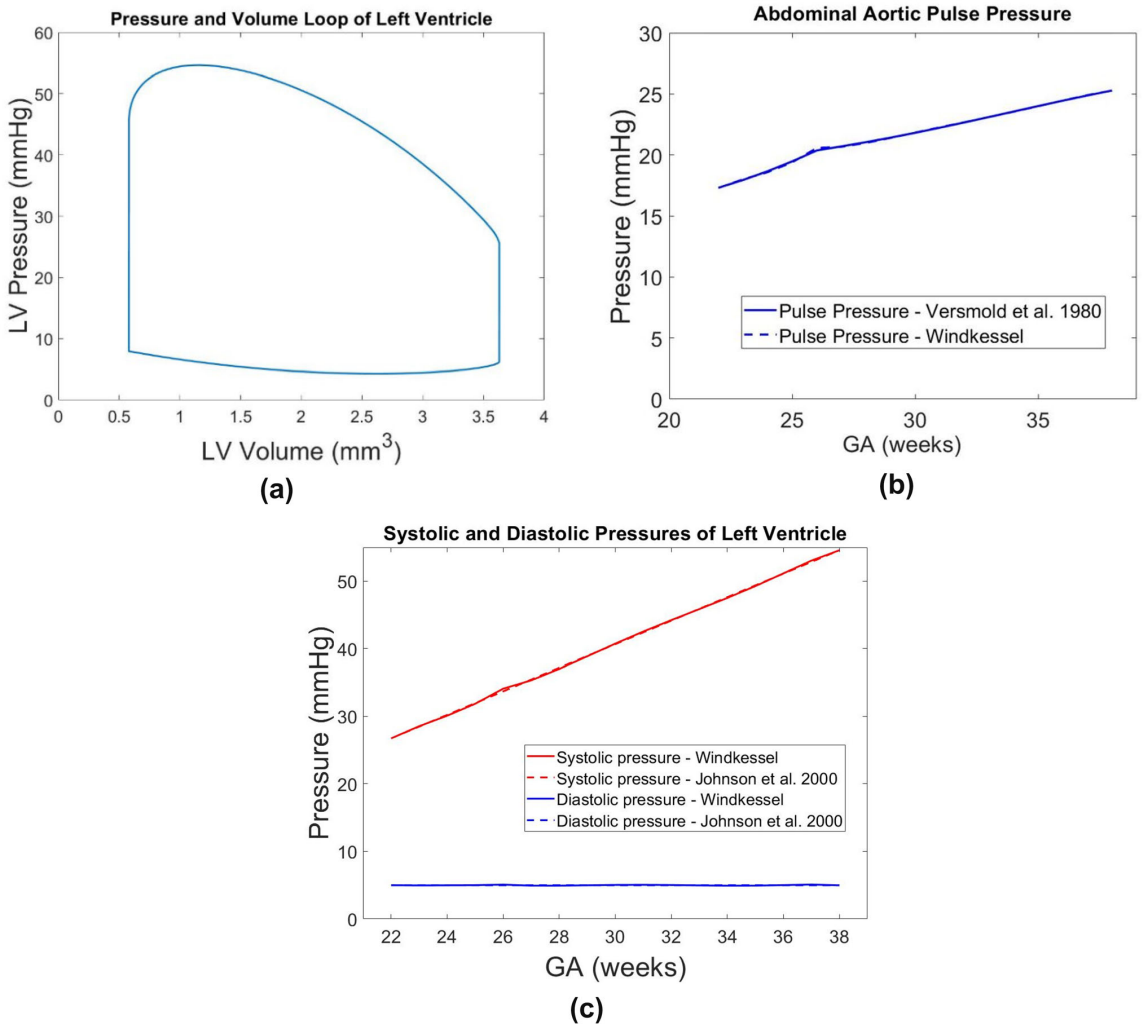
Outputs from our Windkessel lumped parameter model showed physiologic **A** pressure-volume loop and **B** abdominal aortic pulse pressure across gestational age and **C** systolic and diastolic pressures of the left ventricle across gestational age. Literature values of clinically measured abdominal aortic pulse pressure by Versmold *et al.*^34^ and LV pressures by Johnson *et al.*^10^ are plotted in **B** and **C** as well, demonstrating that our model had a good match with these literature values.

The age-dependent scaling factors at specific ages between 22 and 38 weeks gestation were first determined by fitting literature values of LV and RV volume clinical measurements (assuming an idealized sinusoidal volume over time waveform),^8^ the fetal abdominal aortic pulse pressures,^34^ and fetal LV systolic and diastolic pressures^10^ at the matching GA. Subsequently, a 5th order and 6th order polynomial fit were calculated for the resistance scaling factor and the capacitance scaling factor, respectively. We interpreted Johnson *et al.*’s data as indicating that the minimum diastolic LV pressure was 5 mmHg regardless of age, rather than diastolic pressure increasing with age, as the latter would lead to excessively high diastolic pressure when extrapolated to the near-term fetus. Figure 1 showed that a satisfactory match was obtained. Further details of the scaling factors are given in S1 of the supplementary text.

The above lumped parameter model for healthy fetuses was directly adopted in an age-matched way for feHLHS cases, but with the following modification. Firstly, patient-specific volumes over time data were adopted for all cardiac chambers except the right atrium (RA), which was approximated using the atrial length, width and the ellipsoid method.^28^ Secondly, aortic stenosis was modelled by iteratively adjusting the aortic orifice in the CFD simulations to match Doppler measurements. Third, mitral regurgitation was modelled in the CFD simulation for feHLHS cases if present. Fourthly, to emulate elevated LA pressures observed in many fetal aortic stenosis cases, we modelled LA pressure (${P}_{\mathrm{LA}}$) as a linear function of ${V}_{\mathrm{LA}}$ and LA compliance (${C}_{\mathrm{LA}}$), as below, and assigned it as an additional pressure source of the LA in the lumped parameter model.

1 $${P}_{\mathrm{LA}}=\frac{{V}_{\mathrm{LA}}}{{C}_{\mathrm{LA}}}$$

### Energy Dynamics and Wall Shear Stress parameters

From the CFD results, the normalized energy loss across time, *ε*, was given as,

2 $${{\varepsilon}} = \smallint _{T}^{{}} \frac{{Wd\left( t \right) + {\text{KE}}_{{{\text{bulk}}}} + {\text{KE}}_{{{\text{MV}}}} + {\text{KE}}_{{{\text{AV}}}} + {\text{KE}}_{{{\text{regurg}}}} }}{{{\text{SV}}}}dt$$

3 $$Wd\left( t \right) = \smallint _{{{\text{CS}}\left( t \right)}}^{{}} P\left( {\vec{v} \cdot \vec{n}} \right)dA$$

4 $${\text{KE}}_{{{\text{bulk}}}} = \frac{{d\left( {\smallint _{{{\text{CV}}\left( t \right)}}^{{}} \frac{1}{2}\rho |\vec{v}\left( t \right)|^{2} dV} \right)}}{{dt}}$$

5 $${\text{KE}}_{x} = \smallint _{{{\text{CS}}_{x} (t)}}^{{}} \frac{1}{2}\rho |\vec{v}\left( t \right)|^{2} \left( {\vec{v} \cdot \vec{n}} \right)dA,x \in \left\{ {{\text{MV}},{\text{AV}},{\text{regurg}}} \right\}$$

where $Wd\left(t\right)$ was the rate of work done by the ventricle wall, ${\text{KE}}_{{{\text{bulk}}}}$ was the rate of change in kinetic energy of the fluid within the ventricle and ${\text{KE}}_{{{\text{MV}}}}$, ${\text{KE}}_{{{\text{AV}}}}$, and ${\text{KE}}_{{{\text{regurg}}}}$ were the kinetic energy flux through the mitral valve, aortic valve, and mitral regurgitation orifice, respectively, ${\text{CS}}$ was the control surface, ${\text{CV}}$ was the control volume, $A$ was the surface area, $t$ was time, and $T$ was the cardiac cycle duration. Lastly, the oscillatory shear index (OSI) was calculated as,^12^

6 $${\text{OSI}} = \frac{1}{2}\left( {1 - \frac{{\left| {\smallint _{0}^{T} \mathop {{\text{WSS}}}\limits^{ \rightharpoonup } dt} \right|}}{{\smallint _{0}^{T} \left| {\mathop {{\text{WSS}}}\limits^{ \rightharpoonup } } \right|dt}}} \right)$$

where ${\mathop {{\text{WSS}}}\limits^{ \rightharpoonup } }$ is the wall shear stress vector.

### Blood Turnover and Washout Efficiency

To evaluate blood turnover within the LV chamber, we performed simulations with two different fluids, existing blood in the chamber and fresh blood from inflow, using an arbitrary but low diffusivity coefficient 10^−9^ m^2^/s which minimized mixing and enabled better differentiation of the two fluids.

### Diastolic Vortex Formation Time

To further characterize diastolic vorticity, we calculated the formation time (${\tau }^{*}$) of the mitral inflow vortex rings, as,

7 $${\tau }^{*}= \overline{{U/D}}t$$

where $U$ was the mitral inflow velocity, $D$ was the jet diameter, *t* was the duration of either the E or A wave, whichever was higher, or the entire diastolic duration for monophasic flow, and $\overline{{U/D}}$ was the time-averaged ratio between $U$ and $D$.^3^

### Statistical Analysis

All continuous variables were checked for normal distribution using the Anderson-Darling test. A comparison of continuous variables between the two sub-groups (Healthy vs feHLHS) was performed using a *t*-test for independent samples if they had normal distribution or using the Mann-Whitney *U* test if not. Statistical significance was determined at *P* < 0.05.

## Results

### Physiological Features

The peak local valve velocities of our simulations were successfully tuned to achieve a satisfactory match with Doppler velocity measurements at the valves, as demonstrated in Supplementary Table S2. We report a velocity error of − 0.005 ± 0.049 m/s or a percentage error of 4.5% ± 4.3%. Table 2 compared the left heart parameters of the five feHLHS cases used for CFD simulations to the healthy cohort. In the feHLHS cohort, AV velocities were much higher than that in normal LVs, with all cases exceeding the 95th percentile of normal LVs, and 2 cases exceeded twice of this the 95th percentile value. Diastolic E or A wave velocities were also elevated above the 95th percentile of normal LVs, except for feHLHS-2. Mitral inflow was monophasic for 3 cases, and thus no E-wave velocities were recorded. Severe regurgitation was observed, with peak regurgitation velocity ranging from 2.60 to 4.69 m/s. These high velocities translated to a high transmitral pressure gradient of 27–88 mmHg via Bernoulli’s equation, indicating severely elevated pressures, which was likely the reason for the enlarged LV end-diastolic volume (EDV). LV stroke volume (SV) was depressed with varying severity, and given the usually enlarged LV, ejection fraction (EF) was low. In terms of the RV, all feHLHS cases except for feHLHS-3 were moderately enlarged and had higher stroke volumes. Generally, large variability in physiological features was observed amongst feHLHS cases, similar to literature reports.^31^

**Table 2:** Comparison of cardiac parameters between healthy fetuses and case-specific feHLHS.

Parameter	Healthy cohort			Disease cohort						*P* value
	21–22 week (*n* = 6)	28–31 week (*n* = 6)	Overall mean	feHLHS-1, 22.14 week	feHLHS-2, 22.57 week	feHLHS-3, 29.14 week	feHLHS-4, 29 week	feHLHS-5, 31.86 week	Overall mean	
Peak AV velocity, m/s	0.66 ± 0.09 [0.57, 0.75]	0.83 ± 0.12 [0.71, 0.96]	0.74 ± 0.14	1.55	1.79	2.38	1.16	2.68	1.91 ± 0.62	0.009*
Diastolic E-wave, m/s	0.31 ± 0.08 [0.21, 0.40]	0.40 ± 0.09 [0.31, 0.49]	0.33 ± 0.14	0.77	–	–	–	1.31	–	–
Diastolic A-wave, m/s	0.37 ± 0.06 [0.31, 0.44]	0.45 ± 0.09 [0.35, 0.55]	0.41 ± 0.09	0.49	0.38	1.05	0.94	1.45	0.86 ± 0.44	0.12
Diastolic E-wave after intervention, m/s	–	–	–	0.60	–	0.80	0.40	1.10	0.73 ± 0.30	–
Diastolic A-wave after intervention, m/s	–	–	–	0.30	0.65	1.30	0.60	1.20	0.76 ± 0.37	–
Peak MV Regurg., m/s	–	–	–	2.70	3.04	3.15	3.12	4.66	3.33 ± 0.76	–
RV SV, ml	0.59 ± 0.12 [0.46, 0.71]	1.67 ± 0.58 [1.07, 2.27]	1.13 ± 0.57	0.73	1.09	1.56	2.30	4.29	1.99 ± 1.41	0.19
LV SV, ml	0.53 ± 0.16 [0.37, 0.70]	1.92 ± 0.84 [0.88, 2.96]	1.16 ± 0.90	0.17	0.14	1.23	0.68	2.26	0.90 ± 0.88	0.25
LV SV after intervention, ml	–	–	–	0.50	0.16	1.94	1.22	NA	–	–
RV EDV, ml	1.02 ± 0.19 [0.82, 1.22]	3.92 ± 1.14 [2.72, 5.11]	2.47 ± 1.29	1.30	2.91	3.16	5.29	8.28	4.19 ± 2.69	0.25
LV EDV, ml	0.90 ± 0.21 [0.68,1.13]	3.62 ± 1.14 [2.20, 5.03]	2.14 ± 0.16	2.46	2.99	17.42	11.09	8.43	8.48 ± 6.19	0.009*
LV EF, %	59 ± 10 [49, 69]	53 ± 15 [37, 69]	56.2 ± 12.0	6.80	4.63	7.06	6.25	26.8	10.3 ± 9.3	0.009*

Data are presented as mean ± SD and [5th percentile, 95th percentile]. For a healthy cohort, data from the current study were combined with those from Hadi *et al.* ^36^ NA – There was no clear subject specific image scan data

*AV* aortic valve, *MV* mitral valve, *RV* right ventricle, *SV* stroke volume, *LV* left ventricle, *EDV* end-diastolic volume, *EF* ejection fraction, *Regurg* regurgitation

*P < 0.05 comparing all healthy cases to all diseased cases

### Diastolic Flow Patterns

**Figure 2:**
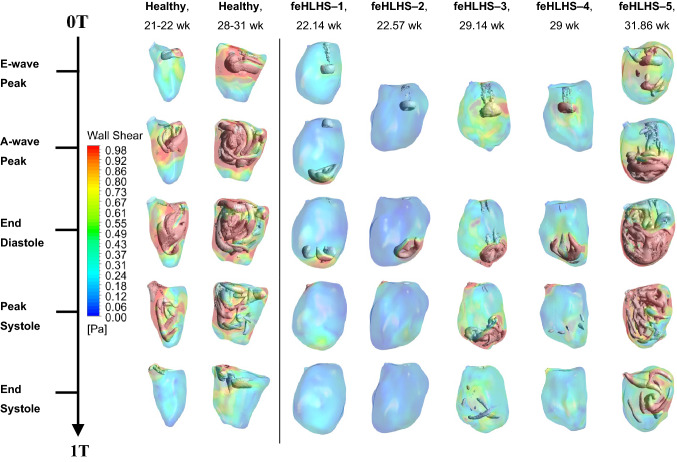
WSS color contour and λ_2_ iso-velocity surfaces in two representative healthy fetal LVs and five feHLHS LVs. These results are also shown in Supplementary Videos S1–10.

Flow patterns within the fetal LVs from CFD simulations are shown in Figure 2, Supplementary Figure S1, and Supplementary Videos S1–10. Vortex dynamics in normal fetal LVs were similar to previous descriptions,^13^ where wide diastolic vortex rings, corresponding to E- and A-wave flow were observed to propagate from the mitral towards the apex. These vortex rings occupied the entire width of the LV and propagated to various extents towards the LV apex. The rings merged and interacted with endocardial walls, bringing about elevated WSS along the way and creating complex secondary vorticity structures that did not dissipate completely but were ejected during the subsequent systole.

Flow patterns in feHLHS were drastically different. Here, MV inflow occurred as a narrow jet with typically high velocity, generating a small-diameter vortex ring that propagated quickly towards the apex and impinged on the apical endocardium. In our simulations, narrowed mitral inlet orifice was necessitated so that the simulation can yield inflow velocities that matched with high Doppler measurements (Supplementary Table S2). This suggested mild MV stenosis, which consequently resulted in the observed fast and narrow inflow jets. Table 3 quantifies the MV annulus and inflow jet dimensions, demonstrating that while inflow was narrowed in feHLHS cases, their MV annulus was not small. This suggested that the MV was not hypoplastic, but rather, it was an impaired ability for MV to open that caused the stenosis. After aortic valvuloplasty intervention, although stroke volume increased, peak MV inflow velocities did not decrease consistently across cases and remained above the 95 percentile of those for normal hearts (Table 2). This suggested that the MV stenosis was due to a malformed valve, rather than congestion of fluid in the LV preventing proper MV opening motions.

**Table 3:** Case-specific mitral valve parameters of healthy LVs and feHLHS LVs.

Parameter	Healthy cohort						Disease cohort						*P* - value
	H1 21 week	H2 21 week	H3 28 week	H4 31 week	H5 31 week	Overall mean	feHLHS-1, 22.14 week	feHLHS-2, 22.57 week	feHLHS-3, 29.14 week	feHLHS-4, 29 week	feHLHS–5, 31.86 week	Overall mean	
MV annulus (mm)	6.60	6.50	8.00	10.5	9.60	8.2 ± 1.8	5.70	13.5	9.50	10.4	10.8	10.0 ± 2.8	0.35
MV annulus (z-score)	0.74	0.62	−0.36	0.80	0.11	0.38 ± 0.50	1.28	− 1.02	3.26	0.63	0.77	0.98 ± 1.54	0.22
MV jet area (mm^2^)	9.00	12.5	40.7	46.4	56.1	32.9 ± 21.0	1.96	3.45	15.0	10.2	14.1	8.9 ± 6.0	0.12
MV jet area/MV annulus (mm)	1.36	3.99	5.09	4.42	5.84	4.14 ± 1.70	0.19	0.61	1.11	1.07	1.30	0.86 ± 0.45	0.009*

Data are presented as mean ± SD

*MV* mitral valve

*P < 0.05 comparing all healthy cases to all diseased cases

Due to chamber dilation and narrow inflow jet, the inflow vortex rings in feHLHS LVs had minimal interaction with the endocardial walls before impinging at the apex. The jet impingement stirred up secondary vortices and elevated wall shear stresses (WSS) around the impingement location. The strength and complexity of secondary vortices and magnitude of WSS depended on the MV inflow velocity. These vortices were weak for feHLHS-1, 2 and 4, and moderate for feHLHS-3, but in feHLHS-5, chaotic flow structures erupted from the high-speed impingement and spread throughout the LV, elevating WSS over a substantial surface area.

Contrary to normal fetal hearts, feHLHS LVs mostly demonstrated monophasic inflow, which was frequently reported for this disease.^15^ Only feHLHS-1 and 5 demonstrated a discernible biphasic mitral inflow profile, which was likely linked to better diastolic ventricular function.^5^ This further contributed to the difference in vorticity pattern complexity between these subjects and the other diseased subjects. Overall, feHLHS LVs had distinctive flow features apart from normal LVs, but there was also considerable variability between individuals within the diseased cohort.

### LV Energy Dynamics

Flow stresses and energy dynamics results from the CFD simulations are provided in Table 4. Systolic intraventricular pressure gradient (IVPG) was calculated as the maximum pressure difference between the apical region of the LV and the aortic outflow boundary during systole, while diastolic IVPG was similarly calculated between the apical region and the mitral inlet during diastole. Unsurprisingly, systolic IVPG was severely elevated in feHLHS LVs compared to normal, due to the outflow stenosis. Diastolic IVPG was also elevated in all cases except for feHLHS-2, due to high inflow velocities from stenotic MV. In feHLHS-2, inflow IVPG was low, due to a low inflow velocity, but this was likely associated with a severely low EF of 4.63%.

**Table 4:** Comparison between healthy LVs and feHLHS LVs derived from CFD simulations.

Parameter	Healthy cohort			Disease cohort						*P* - value
	21-22 week^‡^ (*n* = 6)	28-31 week^‡^ (*n* = 6)	Overall mean	feHLHS-1, 22.14 week	feHLHS-2, 22.57 week	feHLHS-3, 29.14 week	feHLHS-4, 29 week	feHLHS-5, 31.86 week	Overall mean	
Systolic IVPG (Pa)	194 ± 58 [133, 255]	292 ± 91 [197, 387]	242 ± 89	1211	1607	2936	697	3575	2005 ± 1207	0.009*
Diastolic IVPG (Pa)	32 ± 11 [21,44]	51 ± 32 [17, 85]	41 ± 25	91	22	98	105	588	181 ± 230	0.35
Normalised Systolic Ejection Work Done (J/m^3^)	123 ± 33 [88,157]	204 ± 74 [126, 282]	164 ± 69	2256	2846	2765	2534	5351	3150 ± 1251	0.009*
Normalised Systolic Energy Loss (J/m^3^)	22 ± 6 [15, 29]	26 ± 7 [19, 32]	22 ± 8	132	163	212	161	275	188 ± 56	0.009*
Normalised Diastolic Energy Loss (J/m^3^)	16 ± 9 [6, 26]	22 ± 10 [12, 32]	17 ± 10	114	34	167	97	426	168 ± 152	0.009*
Time- & Surface-Ave WSS (Pa)	0.68 ± 0.19 [0.48, 0.87]	0.91 ± 0.27 [0.63, 1.19]	0.77 ± 0.26	0.25	0.15	0.56	0.39	1.50	0.57 ± 0.54	0.14
Diastolic Peak Surface-Ave WSS (Pa)	0.81 ± 0.29 [0.50, 1.12]	1.11 ± 0.39 [0.71, 1.51]	0.90 ± 0.38	0.51	0.18	0.91	0.56	4.04	1.21 ± 1.42	0.46
Diastolic Time- & Surface-Ave WSS (Pa)	0.54 ± 0.16 [0.38, 0.71]	0.81 ± 0.25 [0.55, 1.07]	0.51 ± 0.36	0.27	0.14	0.46	0.38	1.78	0.61 ± 0.67	0.35
Systolic Peak Surface-ave WSS (Pa)	1.41 ± 0.31 [1.08, 1.74]	1.69 ± 0.29 [1.39, 2.00]	1.52 ± 0.36	0.31	0.23	1.03	0.50	2.77	0.97 ± 1.05	0.18
Systolic Time- & Surface-Ave WSS (Pa)	0.85 ± 0.17 [0.67, 1.03]	1.05 ± 0.20 [0.84, 1.26]	0.93 ± 0.22	0.23	0.17	0.62	0.40	1.27	0.54 ± 0.44	0.15

Data are presented as mean ± SD and [5th percentile, 95th percentile]

*IVPG* intraventricular pressure gradient, *Ave* averaged, *WSS* wall shear stress

*P < 0.05 comparing all healthy cases to all diseased cases

^‡^ Data from healthy patient cases combined with Hadi *et al.*^36^

Furthermore, systolic and diastolic energy loss and systolic work done showed a marked increase for all feHLHS cases. Due to aortic stenosis, there was elevated aortic velocity and narrowed outflow channel, leading to high fluid stresses and excessive energy losses in the flow convergence zone of the outlet. A high amount of energy expenditure was thus needed by the LV wall to overcome high frictional energy losses to force fluid through the AV. Diastolic energy losses were substantially elevated from the normal ranges as well. This was likely due to the elevated inflow velocity and the concentration of fluid forces at the impingement point that led to high viscous dissipation. Overall, we observed high-pressure gradients, energy losses and work done in LVs of the feHLHS cohort.

### LV Wall Shear Stress

**Figure 3:**
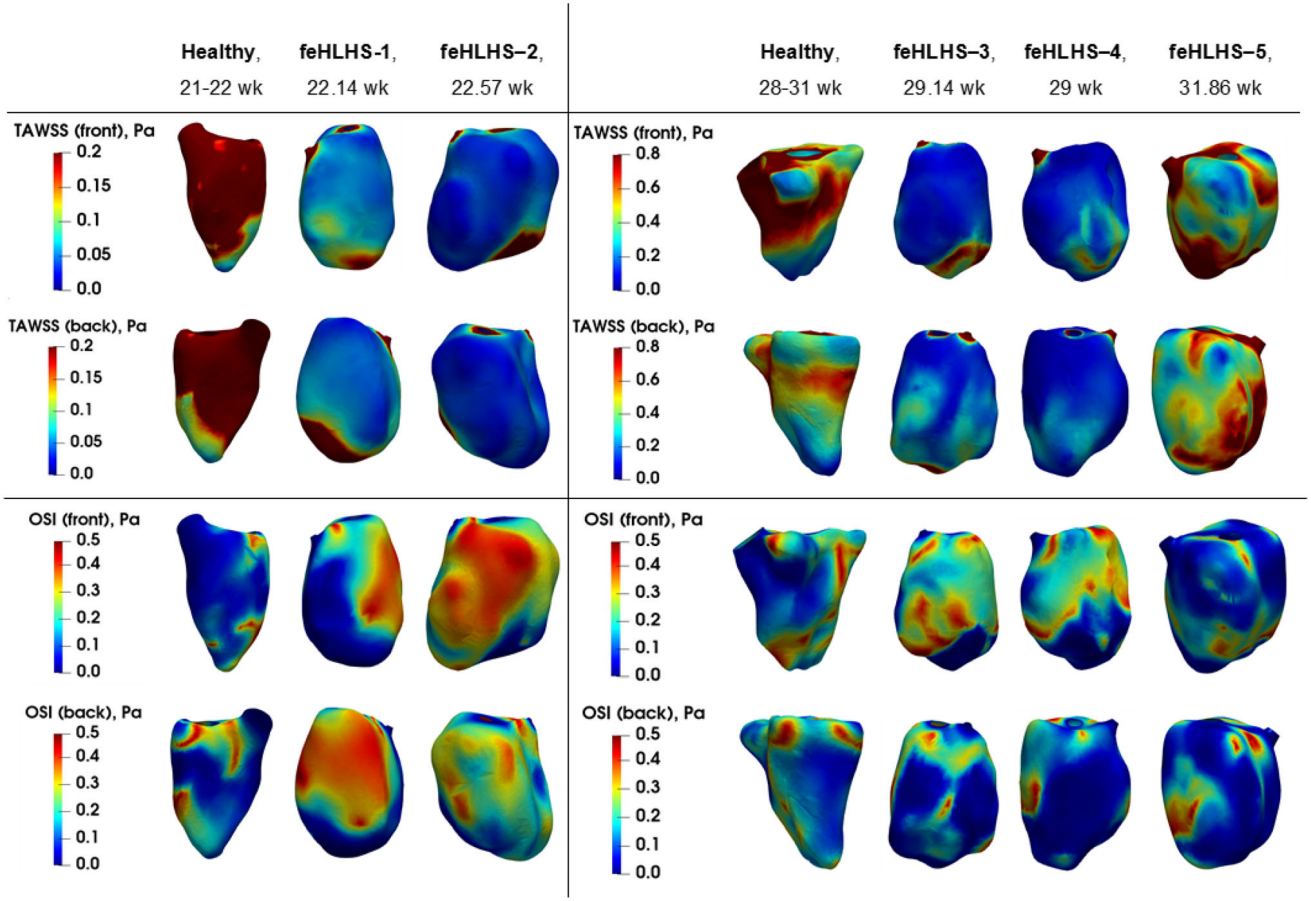
Surface color contour maps of time-averaged WSS (TAWSS) and oscillatory shear index (OSI) distribution in two representative cases for healthy fetal LVs and five feHLHS LV, organised according to GA group.

The spatial distributions of TAWSS and OSI are shown for the feHLHS cohort and two representative cases from the healthy cohort in Figure 3, and other LVs in Supplementary Figure S2. Generally, we observed that regions with high TAWSS had low OSI, and vice versa. In healthy LVs, WSS was generally elevated at the mid-ventricular zone and near the outlet and was low near the apex, as was reported earlier.^13,36^ Mid-ventricular WSS elevation was likely caused by the more significant interaction between the diastolic vortex rings and the endocardial walls in this zone, while outlet WSS elevation was likely caused by the systolic flow convergence at the outlet, where there were elevated velocities within the narrowed flow channel. These zones of high WSS magnitude were also the zones where OSI was low, which demonstrated the consistent directionality of velocities at these walls.

In the feHLHS cohort, high WSS and low OSI were also observed at the outlet region, likely caused by systolic flow convergence as well. however, WSS was generally elevated not at the mid-ventricle zone, but at the site of diastolic inflow jet impingement and its immediate vicinity, near the apical region. With increased strength of the secondary vorticity structures after impingement, such as in the case of feHLHS-5, WSS elevations could extend further up the LV towards the base. OSI was observed to be low at this impingement point but was higher elsewhere in the LV.

### Turnover of Fluid Within LV

**Figure 4:**
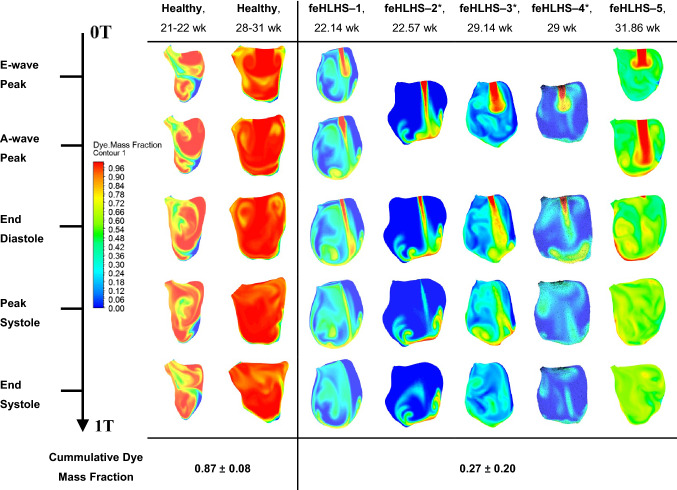
In-plane contour maps of passive dye mass fraction across the cardiac cycle after 3 cardiac cycles for 2 representative cases of healthy LVs and five feHLHS LVs. Mass fraction of 1 (red) indicates ‘fresh’ blood entering from the mitral valve and mass fraction of 0 (blue) represents ‘old’ blood initially present in the ventricle. *feHLHS-2,3,4 has a monophasic inflow profile. Simulation results for all 5 healthy cases are given in Supplementary Figure S3.

**Figure 5:**
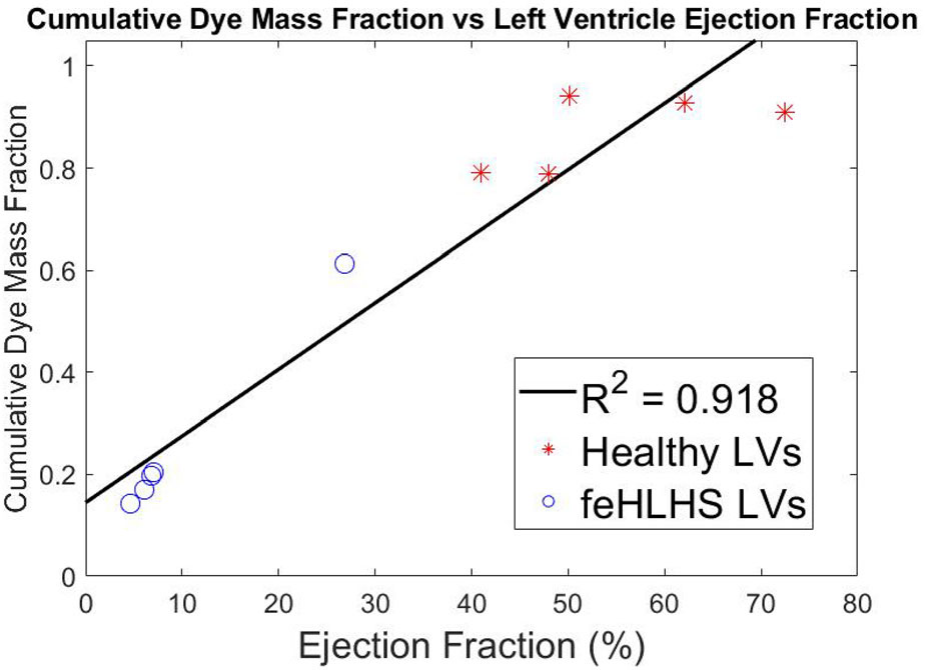
Scatterplots evaluating the volume-averaged cumulative dye mass fraction after three cardiac cycles vs. left ventricle ejection fraction (%)

Figure 4 and Supplementary Figure S3 plots the color contours of the turnover mass fraction, which was the ratio of freshly introduced blood to initially present blood at the end of 3 cardiac cycles, with red indicating a high fraction of freshly introduced blood, and blue indicating a low fraction. It was clear that the turnover of blood in the LVs of feHLHS was much lower than that in normal LVs, with the volume-averaged cumulative dye mass fraction of normal LVs being approximately three times that of stenotic LVs. Turnover was lowest at the apex for normal LVs, but in stenotic LVs, turnover was the highest at the apex, due to the high-velocity inflow jet that propelled incoming fluid quickly towards the apex. Furthermore, turnover appeared to depend on EF, as shown in the regression analysis in Fig. 5.

### Diastolic Vortex Formation Time

Table 5 shows the vortex formation time for all simulation cases.

**Table 5:** Case-specific vortex formation times obtained from CFD simulations.

Parameter	Healthy cohort					Disease cohort				
	H1 21 week	H2 21 week	H3 28 week	H4 31 week	H5 31 week	feHLHS-1 22.14 week	feHLHS-2 22.57 week	feHLHS-3 29.14 week	feHLHS-4 29 week	feHLHS–5 31.86 week
Formation time	9.3	6.84	3.5	2.3	4.7	15.1	25.9	18.4	19.4	15.1

## Discussion

We conducted image-based flow simulations in LVs of feHLHS, to understand essential fluid dynamics differences from normal LVs. Our cohort of disease patients was from a pool of patients fitting the selection criteria for FAV and eventually underwent the intervention. Our focus on this cohort of patients is due to evidence that such interventions have the potential of improving medium-term survival for the cohorts,^20,23^ and an improved understanding of disease fluid dynamics may lead to optimization of the intervention.

We find that in the disease cohort, there was substantial variability in flow patterns and forces, but there were unifying features. The most distinctive feature in the feHLHS cohort was that of a narrow and fast inflow jet that moved quickly from the mitral inlet to impinge on the apical region, which led to excessive dynamics at the apical region but allowed the basal region to be more quiescent. In contrast, in normal LVs, the inflow vortices were slower and interacted more substantially with the mid-ventricular endocardial walls, leading to elevated vorticity dynamics at the mid-ventricular and basal region, but more quiescent flow at the apex.

The elevation of mitral inflow velocity was somewhat surprising, as one would expect the high LV pressure in the diseased LV to oppose mitral inflow and lead to decreased rather than increased inflow velocities. Our analysis suggested that this elevated inflow velocity was not due to a hypoplastic mitral annulus but was more likely associated with the impaired mitral valve opening dynamics because the inflow jet diameter was small despite a normal-sized mitral annulus. Since these velocities did not show acute improvement after aortic valvuloplasty intervention, it was likely that the mitral valve was malformed and had impaired opening function. However, further studies to validate this notion and understand the details are warranted.

Since the mitral inflow was observed to be abnormal, we computed the formation time of diastolic inflow vortex rings (Table 5), as this was previously proposed to be capable of classifying abnormal LV fluid dynamics.^7^ We noted with interest that the vortex formation times in healthy LVs were close to that reported in normal adult hearts, which had a range of between 2 and 7.^6^ This suggested similar vortex characteristics between pre-and post-natal LVs. In feHLHS cases, however, formation times were considerably higher, due to greater blood inflow velocities and smaller MV diameters. This suggests that diastolic vortex generation in feHLHS LVs was energy inefficient, which corroborated with observations of higher diastolic energy losses.

A second distinctive feature of feHLHS LV was that both the mitral inflow and the aortic outflow were associated with elevated IVPG and energy losses. Aortic outflow energy losses per unit volume of flow were found to be one order of magnitude higher, but this was not surprising, given the severity of the stenosis. These excessive energy losses seemed likely to impose an excessive burden on the LV and could be contributing factors to the progression of HLHS at birth.

The third distinctive feature was a reduced blood turnover in feHLHS LVs, where the renewal of blood within the LV was 66% slower on the average. This was likely to induce hypoxic stress in the endocardium. In a chick embryonic model of HLHS induced by left atrial ligation, histology demonstrated increased regions of hypoxia consequent to altered LV hemodynamics, and at the same time, elevated deposition of collagen I. This bore similarity to clinical endocardial fibroelastosis (EFE) that could often be observed in feHLHS LVs as hyper-echogenic endocardium.^19,22^ A plausible mechanism for this was the hypoxia-induced endothelial-to-mesenchymal transition (EndMT), as fibroelastosis was shown to be related to EndMT,^40^ which hypoxia was shown to induce.^41^

EFE has been associated with diastolic dysfunction in feHLHS. LVs with severe EFE are associated with bad diastolic dysfunction and greater impairment in LV growth after FAV,^5,19^ and as such is important to understand. A previous study suggested that flow disturbances such as stagnant intracavity flow and aortic regurgitation led to the development of EFE.^26^ Our disease LVs matched this description, having stenotic mitral inflow. While WSS patterns were qualitatively different in feHLHS cohort, we did not find consistent trends in the overall spatially-averaged WSS magnitude, due to large variability between cases, which dampened the notion that specific WSS characteristics could have caused EFE or impairment on the growth of the left heart. However, further investigations on these issues seemed warranted here as well.

Since the overall WSS magnitude did not demonstrate clear differences between normal and feHLHS LVs, we suggest that this may not be a consequential factor in determining the progression of feHLHS LVs towards HLHS at birth. It seemed more likely that the drastic elevation of LV pressure and thus stresses on the myocardium and valves, and the lack of myocardial deformational motion and leaflet dynamics were more closely related to the lack of growth of these cardiac structures.

Further, it seemed that some of the essential differences between feHLHS and healthy LVs were resolvable with FAV. In the post-intervention data from our patient cohort, a large proportion of fetuses developed a biphasic mitral inflow profile and improved stroke volumes as demonstrated in Table 2, which was likely to alter the fluid patterns and resolve the hypoxic and hypertensive stresses, and the high work burden on the heart. However, aortic regurgitation was common in post-intervention LVs.^2,17^ The post-intervention LV fluid dynamics was thus likely to be quite different from both the pre-intervention feHLHS LV, and the normal LV, and detailed investigation seemed warranted.

Our current study provides a baseline disease characterization, such that when post-intervention fluid dynamics data is available, we can compare pre-and post-intervention to understand the effects of the intervention. We can also use the same approach to understand the effects of growth and remodelling changes to cardiac fluid dynamics, such as if there was a regression of the aortic stenosis after the intervention or weakening of cardiac contractility, and we can also study the relationship between fluid mechanical stimuli and cardiac growth and gestational development. All of these may lead to an improved understanding of the effects of the intervention and of how the flow of mechanical stimuli affects outcomes, which can inform clinical decisions such as the suitability of specific patients for the intervention, when to conduct the intervention, and whether novel medical devices such as fetal transcatheter replacement aortic valve can be beneficial.

There are some limitations to our study. Firstly, our simulations did not account for the fluid-structure interaction between fluid and the mitral valve and were simplified as an effective inlet orifice area, due to an inability to observe valve morphology as images had poor resolution and high noise. This was likely to have altered the resulting flow patterns, as the presence of the mitral valve would increase vortex penetration, wall shear stress and viscous dissipation.^33^ However, the main flow phenomena such as the inflow jet vortex ring and its interaction with ventricular walls were likely to be retained. Secondly, we approximated the locations for the valve orifices as we could not imagine their exact location, and this could have led to errors. Thirdly, due to limited imaging resolution and image noise, there are likely to be some errors in our segmentation and cardiac motion estimation.

In conclusion, we performed patient-specific 4D flow simulations of normal and feHLHS LVs and found essential differences. There was wide variability observed in feHLHS cases, but also unifying features. Firstly, in feHLHS LVs, an abnormally fast and narrow diastolic mitral inflow jet was observed, likely due to a malformed mitral valve with impaired ability to open. This inflow jet impinged at the apex, causing elevated vorticity dynamics and WSS at the apex region, rather than the mid-ventricle region as was the case for normal LV. Secondly, in feHLHS LV, there were further elevated intraventricular pressure gradients, and higher systolic and diastolic energy losses, due to both AV and MV stenosis. Thirdly, feHLHS LV had low blood turnover, suggesting a hypoxic environment, which may be linked to EFE that is frequently observed in such patients.

## Supplementary Information

Below is the link to the electronic supplementary material.

Supplementary file1 (DOCX 591 KB)

Supplementary file2 (MP4 290 KB)

Supplementary file3 (MP4 436 KB)

Supplementary file4 (MP4 543 KB)

Supplementary file5 (MP4 640 KB)

Supplementary file6 (MP4 1034 KB)

Supplementary file7 (MP4 742 KB)

Supplementary file8 (MP4 1072 KB)

Supplementary file9 (MP4 1675 KB)

Supplementary file10 (MP4 1141 KB)

Supplementary file11 (MP4 1077 KB)
