# Supplementary material for: Fluid Mechanics of Fetal Left Ventricle During Aortic Stenosis with Evolving Hypoplastic Left Heart Syndrome
Source: Ann Biomed Eng. 2022 Jun 22;50(9):1158–72. doi: 10.1007/s10439-022-02990-5 (PMC9363377; doi:10.1007/s10439-022-02990-5)
Supplement: Supplementary file 1 — Supplementary file1 (DOCX 591 KB) [file 10439_2022_2990_MOESM1_ESM.docx]

**Fluid Mechanics of Fetal Left Ventricle During Aortic Stenosis with Evolving Hypoplastic Left Heart Syndrome**

Hong Shen Wong^1^*, Hadi Wiputra^2^*, Andreas Tulzer^3^, Gerald Tulzer^3^, Choon Hwai Yap^1^

1. Department of Bioengineering, Imperial College London, UK
2. Department of Biomedical Engineering, University of Minnesota, USA
3. Department of Pediatric Cardiology, Children’s Heart Center Linz, Kepler University Hospital, Linz, Austria

* Authors have equal contributions

###### Section S1. Additional Details on Lumped Parameter Model.

The methodology for allometric scaling of the lumped parameter model from 38 weeks GA to other GA was obtained from Pennati et al. ^3^, and is described as,

𝑊𝐺𝐴 −1

𝑅𝐺𝐴 = 𝑅38 (𝑊38 )

(1)

𝑊𝐺𝐴

1.33

𝐶𝐺𝐴 = 𝐶38 (𝑊38 )

(2)

𝑊𝐺𝐴

−0.33

𝐿𝐺𝐴 = 𝐿38 (𝑊38 )

(3)

𝑊𝐺𝐴

−1.33

𝐾𝐺𝐴 = 𝐾38 (𝑊38 )

(4)

log_10_ 𝑊_𝐺𝐴_ = 0.2508 + 0.1458 𝐺𝐴 − 0.0016 𝐺𝐴^2^ (5)

where 𝑅_𝐺𝐴_, 𝐶_𝐺𝐴_, 𝐿_𝐺𝐴_ and 𝐷_𝐺𝐴_ were the resistance, compliance, inertances, and valvular dissipative coefficient of an element in the lumped parameter model at the gestational age (GA), respectively. We rescaled Pennati et al.’s model at various GA between 22 and 38 weeks to enable a match to literature values of LV and RV volume clinical measurements,^1^ fetal abdominal aortic pulse pressures,^4^ and fetal LV systolic and diastolic pressures.^2^ This was achieved via a resistance scaling factor to all resistances in the model, 𝑅_𝑠𝑐𝑎𝑙𝑒_, and a compliance scaling factor to all compliances in the model, 𝐶_𝑠𝑐𝑎𝑙𝑒_. Subsequently, a 5^th^ or 6^th^ order polynomial fit was performed to describe 𝑅_𝑠𝑐𝑎𝑙𝑒_ and 𝐶_𝑠𝑐𝑎𝑙𝑒_ as functions of GA, as given in equations S6 and S7 and table S1. Two separate 5^th^ order polynomials were needed for the compliance scaling for a good fit, delineated at GA of 27wk, due to a transition in 𝐶_𝑠𝑐𝑎𝑙𝑒_ trends with GA at this age.

𝑅_𝑠𝑐𝑎𝑙𝑒_ = 𝐴_𝑟_ ∗ 𝐺𝐴^6^ + 𝐵_𝑟_ ∗ 𝐺𝐴^5^ + 𝐶_𝑟_ ∗ 𝐺𝐴^4^ + 𝐷_𝑟_ ∗ 𝐺𝐴^3^ + 𝐸_𝑟_ ∗ 𝐺𝐴^2^ + 𝐹_𝑟_ ∗ 𝐺𝐴 + 𝐺_𝑟_ (6)

𝐶_𝑠𝑐𝑎𝑙𝑒_ = 𝐵_𝑐_ ∗ 𝐺𝐴^5^ + 𝐶_𝑐_ ∗ 𝐺𝐴^4^ + 𝐷_𝑐_ ∗ 𝐺𝐴^3^ + 𝐸_𝑐_ ∗ 𝐺𝐴^2^ + 𝐹_𝑐_ ∗ 𝐺𝐴 + 𝐺_𝑐_ (7)

***Supplementary Table S1:*** *Coefficients for Eq. (6) and Eq. (7) used for the polynomial*

*resistance and capacitance scaling factors.*

| ***Coefficients*** | 𝑹𝒔𝒄𝒂𝒍𝒆 | 𝑪_𝒔𝒄𝒂𝒍𝒆_ (𝒃𝒆𝒍𝒐𝒘 𝟐𝟕𝒘𝒌) | 𝑪_𝒔𝒄𝒂𝒍𝒆_ (𝒂𝒇𝒕𝒆𝒓 𝟐𝟕𝒘𝒌) |
| --- | --- | --- | --- |
| A, 𝑚𝑚𝐻𝑔 𝑠 𝑚𝑙^−1^𝑤𝑘^−6^ | −7.316 ∗ 10^−7^ | − | − |
| B, 𝑚𝑚𝐻𝑔 𝑠 𝑚𝑙^−1^𝑤𝑘^−5^ | 1.336 ∗ 10^−4^ | 5.259 ∗ 10^−7^ | 2.183 ∗ 10^−4^ |
| C, 𝑚𝑚𝐻𝑔 𝑠 𝑚𝑙^−1^𝑤𝑘^−4^ | 1.009 ∗ 10^−2^ | 9.465 ∗ 10^−5^ | −0.0261 |
| D, 𝑚𝑚𝐻𝑔 𝑠 𝑚𝑙^−1^𝑤𝑘^−3^ | 0.403 | 6.85 ∗ 10^−3^ | 1.247 |
| E, 𝑚𝑚𝐻𝑔 𝑠 𝑚𝑙^−1^𝑤𝑘^−2^ | 8.978 | 0.2489 | -29.74 |
| F, 𝑚𝑚𝐻𝑔 𝑠 𝑚𝑙^−1^𝑤𝑘^−1^ | 105.7 | 4.529 | 354.4 |
| G, 𝑚𝑚𝐻𝑔 𝑠 𝑚𝑙^−1^ | 513.9 | −32.79 | −1688 |

###### Section S2. Additional Results

***Supplementary Table S2:*** *Case-specific comparison between computational fluid dynamics simulation result and clinical measurements of valve velocities. In several disease cases and one healthy case, mitral inflow was detected by Doppler to be monophasic, and the only peak inflow velocity data was recorded as the peak A-wave velocity and peak E-wave velocity was left blank. Further mitral regurgitation was only observed in diseased cases.*

***Healthy Cohort Disease Cohort***

| H1  (21 wk) | | | H2  (21 wk) | | H3  (28 wk) | | H4  (31 wk) | | H5  (31 wk) | | feHLHS-1 (22.14 wk) | | feHLHS-2 (22.57 wk) | | feHLHS-3 (29.14 wk) | | feHLHS-4  (29 wk) | | feHLHS–5 (31.86 wk) | |  |
| --- | --- | --- | --- | --- | --- | --- | --- | --- | --- | --- | --- | --- | --- | --- | --- | --- | --- | --- | --- | --- | --- |
| ***Parameter*** | CFD | Dop. | CFD | Dop. | CFD | Dop. | CFD | Dop. | CFD | Dop. | CFD | Dop. | CFD | Dop. | CFD | Dop. | CFD | Dop. | CFD | Dop. | ***P*** |
| Peak AV, m/s | 0.65 | 0.68 | 0.65 | 0.65 | 0.77 | 0.80 | 0.91 | 0.82 | 0.82 | 0.80 | 1.67 | 1.65 | 1.71 | 1.80 | 2.47 | 2.45 | 1.20 | 1.13 | 2.79 | 2.73 | 0.007 |
| Diastolic E, m/s |  |  | 0.30 | 0.30 | 0.46 | 0.40 | 0.59 | 0.60 | 0.40 | 0.40 | 0.76 | 0.83 |  |  |  |  |  |  | 1.31 | 1.30 |  |
| Diastolic A, m/s | 0.41 | 0.45 | 0.48 | 0.45 | 0.47 | 0.45 | 0.35 | 0.38 | 0.46 | 0.55 | 0.49 | 0.53 | 0.37 | 0.37 | 1.20 | 1.10 | 0.93 | 0.85 | 1.37 | 1.40 | 0.031 |
| Peak Regurgitation, m/s |  |  |  |  |  |  |  |  |  |  | 2.70 | 2.60 | 2.79 | 2.60 | 3.24 | 3.20 | 3.11 | 3.13 | 4.65 | 4.69 |  |

***Supplementary Figure S1:*** *WSS color contour and λ2 iso-velocity surfaces in five healthy fetal LVs*


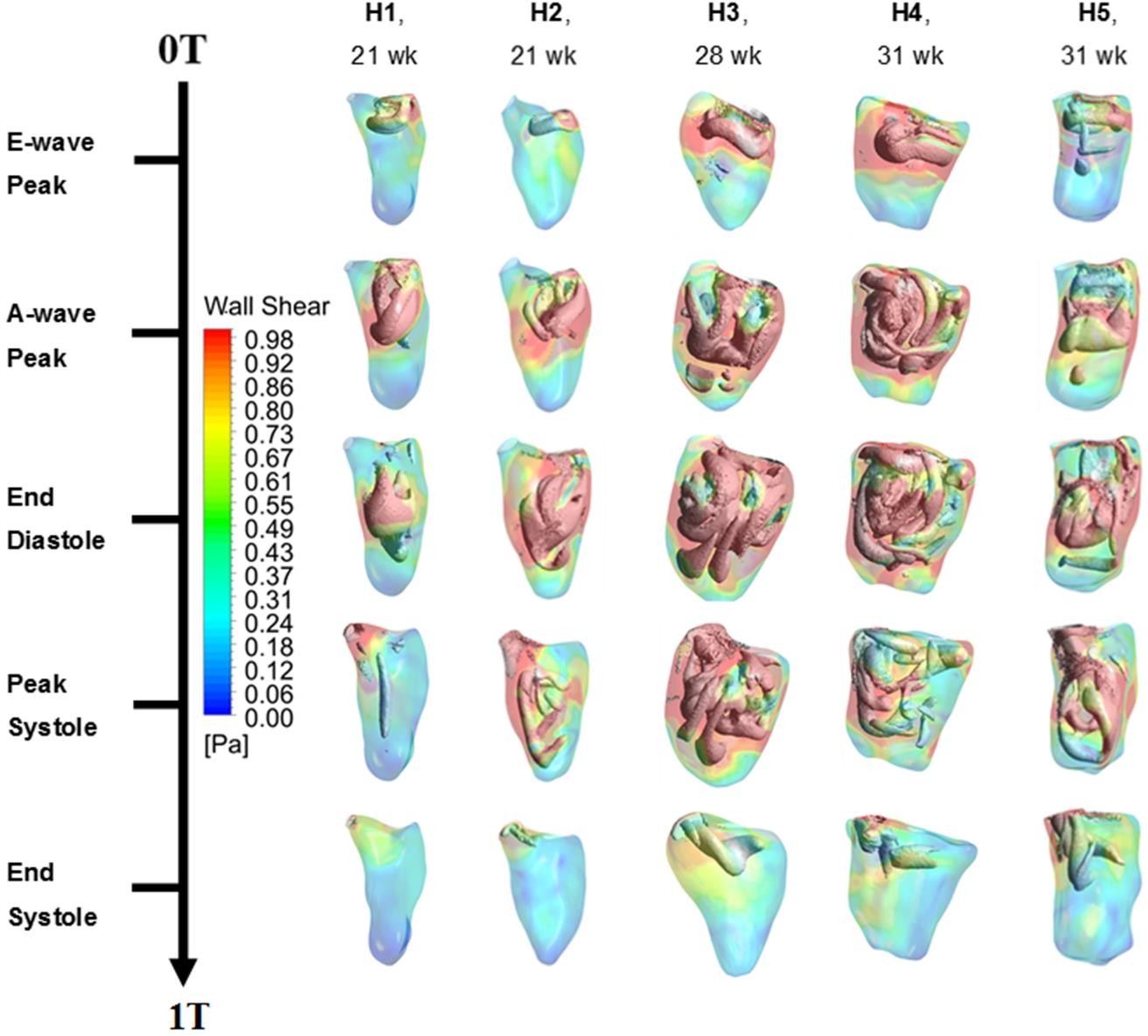


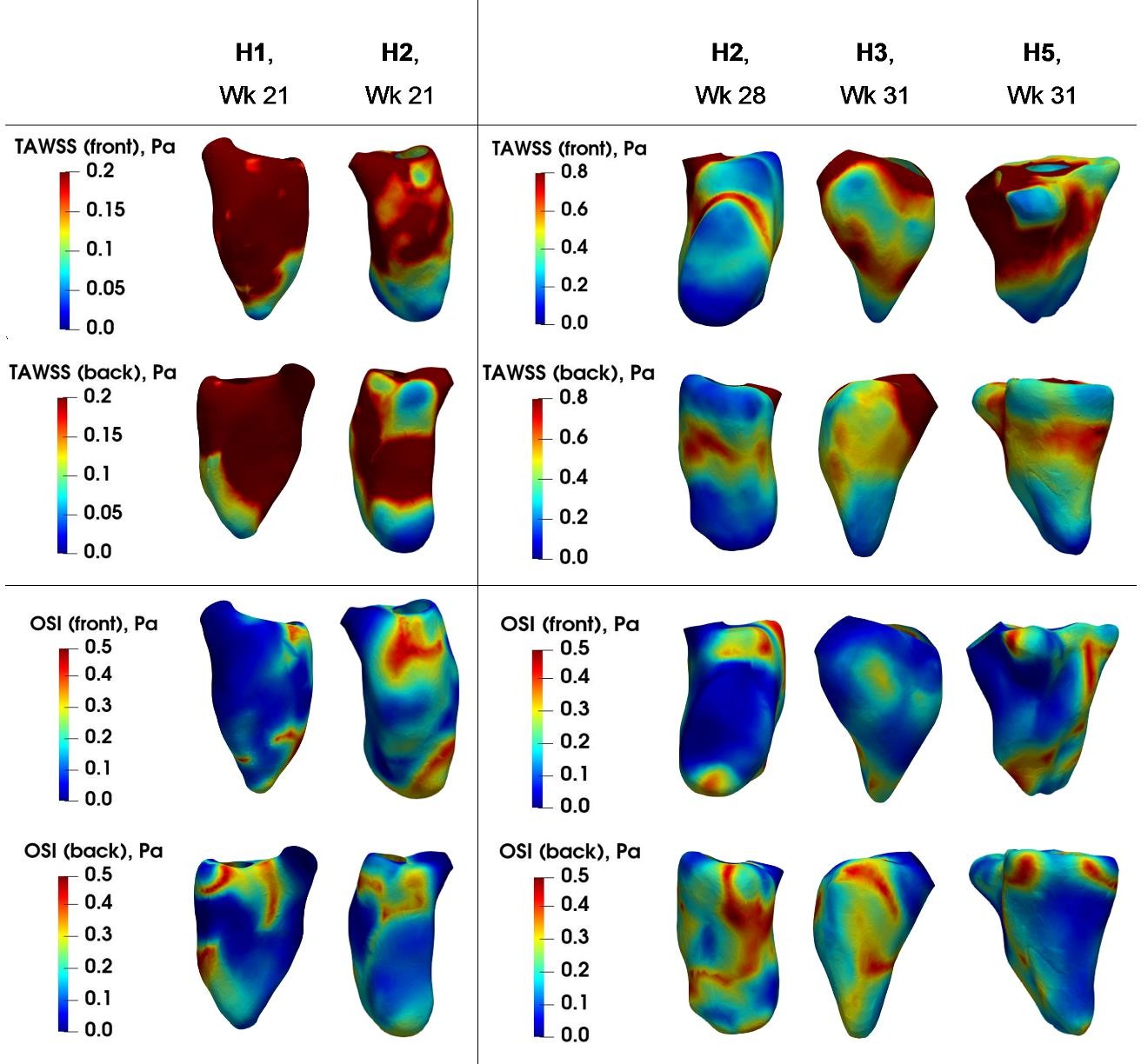
***Supplementary Figure S2:*** *Surface colour contours maps of time-averaged WSS (TAWSS) and oscillatory shear index (OSI) distribution for the 5 healthy fetal LVs*

***Supplementary Figure S3:*** *In-plane contour maps of passive dye mass fraction across the cardiac cycle for 5 healthy LVs. Mass fraction of 1 (red) indicates ‘fresh’ blood entering from the mitral valve and mass fraction of 0 (blue) represents ‘old’ blood initially present in the ventricle.*


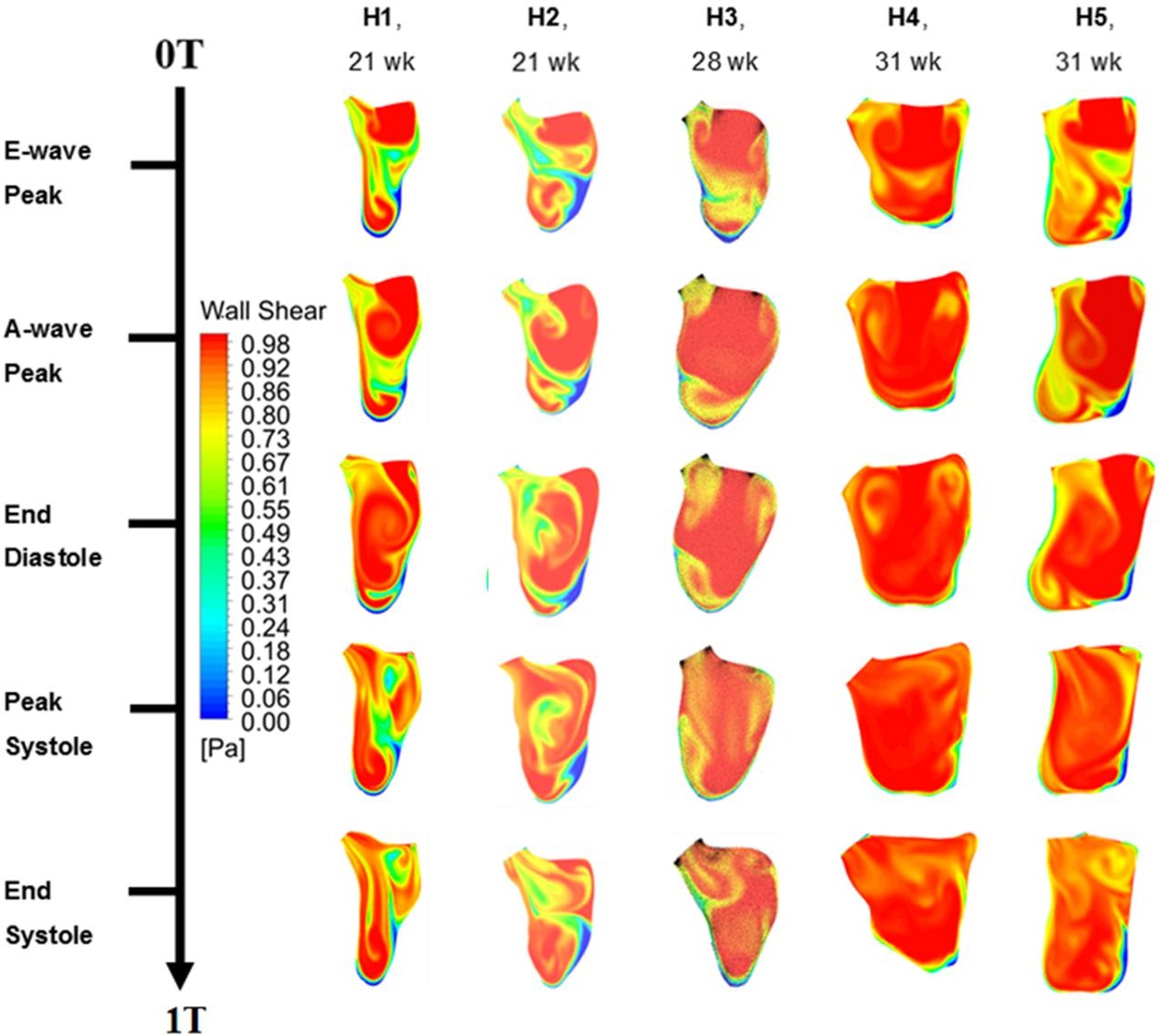


###### References

1. Hamill N., L. Yeo, R. Romero, S. S. Hassan, S. A. Myers, P. Mittal, J. P. Kusanovic, M. Balasubramaniam, T. Chaiworapongsa, E. Vaisbuch, J. Espinoza, F. Gotsch, L. F. Goncalves and W. Lee. Fetal cardiac ventricular volume, cardiac output, and ejection fraction determined with 4- dimensional ultrasound using spatiotemporal image correlation and virtual organ computer- aided analysis. *American Journal of Obstetrics and Gynecology* 205: 76.e71-76.e10, 2011.
2. Johnson P. Intracardiac pressures in the human fetus. *Heart* 84: 59-63, 2000.
3. Pennati G. and R. Fumero. Scaling approach to study the changes through the gestation of human fetal cardiac and circulatory behaviors. *Annals of Biomedical Engineering* 28: 442-452, 2000.
4. Versmold H. T., J. A. Kitterman, R. H. Phibbs, G. A. Gregory and W. H. Tooley. Aortic Blood Pressure During the First 12 Hours of Life in Infants with Birth Weight 610 to 4,220 Grams. *PEDIATRICS* 67: 607-613, 1981.
